# Non-histologic factors discriminating proliferative lupus nephritis from membranous lupus nephritis

**DOI:** 10.1186/s13075-020-02223-x

**Published:** 2020-06-09

**Authors:** Oh Chan Kwon, Jung Hwan Park, Hyeong-Cheon Park, Seung Min Jung, Sang-Won Lee, Jason Jungsik Song, Yong-Beom Park, Min-Chan Park

**Affiliations:** 1 Division of Rheumatology, Department of Internal Medicine, Yonsei University College of Medicine, Seoul, South Korea; 2 Division of Nephrology, Department of Internal Medicine, Yonsei University College of Medicine, Seoul, South Korea

**Keywords:** Systemic lupus erythematosus, Lupus nephritis, Proliferative, Membranous

## Abstract

**Background:**

To investigate non-histologic factors that can discriminate proliferative lupus nephritis (LN) from membranous LN in patients with systemic lupus erythematosus with renal manifestations.

**Methods:**

Patients with biopsy-proven proliferative LN (class III ± V and class IV ± V) and membranous LN (class V) were included. Non-histologic factors were compared between the two groups. A logistic regression analysis was performed to identify the factors associated with proliferative LN. To assess the accuracy of these factors in discriminating between proliferative LN and membranous LN, we performed a receiver-operating characteristic analysis.

**Results:**

Of the total 168 patients with biopsy-proven LN, 150 patients (89.3%) had proliferative LN, and 18 patients (10.7%) had membranous LN. In the multivariable logistic regression analysis, positive anti-double-stranded DNA (anti-dsDNA) antibody (adjusted OR = 11.200, 95% CI = 2.202–56.957, *p* = 0.004) was associated with proliferative LN, while positive anti-U1RNP antibody (adjusted OR = 0.176, 95% CI = 0.040–0.769, *p* = 0.021) and higher glomerular filtration rate (GFR) (adjusted OR = 0.973, 95% CI = 0.951–0.994, *p* = 0.013) were inversely associated with proliferative LN. Among these covariates, the anti-dsDNA antibody (area under the curve = 0.806, 95% CI = 0.695–0.916) had the highest accuracy in discriminating between proliferative LN and membranous LN.

**Conclusion:**

The positivity of anti-dsDNA antibody was associated with proliferative LN, while the positivity of anti-U1RNP antibody and GFR were inversely associated with proliferative LN. The anti-dsDNA antibody had a good accuracy in discriminating proliferative LN from membranous LN.

## Background

Lupus nephritis (LN) is one of the common manifestations of systemic lupus erythematosus (SLE) that causes significant morbidity and mortality [1]. According to the International Society of Nephrology/Renal Pathology Society 2003 classification, LN is classified into six classes according to the glomerular pathology [2]. Among these classes, class III, class IV, and class V have the potential to cause long-term renal damage [3]. Class III and class IV (proliferative LN) are highly inflammatory with immune complex deposition in the subendothelial space, whereas class V (membranous LN) is less inflammatory with immune complex deposition in the subepithelial space [2]. Proliferative LN is usually treated with potent immunosuppressive agents, whereas membranous LN may be managed conservatively with antiproteinuric agents if patients have subnephrotic proteinuria or with immunosuppressive agents if patients have nephrotic-range proteinuria [4, 5]. The risk of progression to end-stage renal disease (ESRD) differs between proliferative LN and membranous LN, with proliferative LN having a worse prognosis (risk of ESRD, 10–20% for proliferative LN vs < 10% for membranous LN) [4, 6]. Considering the differences in the treatment strategy and renal prognosis between proliferative LN and membranous LN, it is important to distinguish one from the other.

The confirmative modality for diagnosing LN and distinguishing proliferative LN from membranous LN is renal biopsy [5]. The American College of Rheumatology (ACR) recommends renal biopsies in patients with SLE who have increasing serum creatinine levels of an unknown cause, proteinuria at protein levels of ≥ 1 g per day (either in a 24-h urine specimen or on a spot protein/creatinine ratio [PCR]), or a combination of the following: proteinuria at protein levels of ≥ 0.5 g per day plus ≥ 5 red blood cells (RBCs) per high power field (HPF) or proteinuria at protein levels of ≥ 0.5 g per day plus cellular casts [7]. Although it is apparently important to perform renal biopsies to confirm the diagnosis of LN and to guide appropriate therapeutic decision-making based on the classification of LN, there are some circumstances where renal biopsies are difficult to perform, such as in patients under mechanical ventilation who have difficulty in assuming the prone position, patients with uncorrectable bleeding diathesis, and patients with small kidney sizes. Given that renal biopsies may not always be available, it is clinically meaningful to identify non-histologic factors that can discriminate proliferative LN from membranous LN.

To date, data regarding clinical factors predictive of proliferative LN are limited. In this study, we aimed to identify non-histologic factors predictive of proliferative LN.

## Materials and methods

### Patients

Data from two independent LN cohorts from two tertiary referral hospitals in Seoul, Korea, were retrospectively reviewed. Both cohorts consisted of patients diagnosed with LN via renal biopsy between July 2006 and December 2018. All patients met the 1997 ACR classification criteria for SLE [8]. Renal biopsies were performed in accordance with the indications recommended by the ACR [7]. The patients were categorized into the proliferative LN (class III, class IV, class III + V and class IV + V) and membranous LN (class V) groups based on their renal biopsy reports. Given that the therapeutic strategy is similar between pure proliferative LN (class III and class IV) and mixed proliferative LN (class III + V and class IV + V), and that the therapeutic strategy in both is different from that in membranous LN [7, 9], pure proliferative LN (class III and class IV) and mixed proliferative LN (class III + V and class IV + V) were both categorized as proliferative LN in the primary analysis. As the purpose of this study was to identify the factors that discriminate proliferative LN from membranous LN, patients with class I, class II, and class VI were excluded. This study was approved by the Institutional Review Board (IRB) of Gangnam Severance Hospital (IRB No: 3-2019-0072). Owing to the retrospective nature of this study, the requirement for informed consent was waived.

### Covariates

Data on the following covariates at the time of renal biopsy were collected: age, sex, presence of hypertension and diabetes mellitus, manifestations of SLE other than those of LN, positivity of antibodies (Abs) to extractable nuclear antigens, anti-double-stranded DNA (anti-dsDNA) Ab, lupus anticoagulant, anti-β_2_ glycoprotein Ab and anti-cardiolipin Ab, C3 and C4 levels, serum albumin and creatinine levels, glomerular filtration rate (GFR), urine PCR, urinalysis results, and SLE Disease Activity Index 2000 (SLEDAI-2 K) [10]. Autoantibodies were measured using an automated fluoroimmunoassay analyzer (EliA; Phadia, Uppsala, Sweden). Lupus anticoagulants were assessed using the IL Test TM LAC Screen/Confirm Kit (Instrumentation Laboratory Co., Bedford, MA, USA).

### Statistical analysis

The patients’ characteristics were summarized using descriptive statistics. To compare the characteristics between the proliferative LN group and membranous LN group, Student’s *t* test or Mann-Whitney test was used for continuous variables and Fisher’s exact test or chi-square test (when appropriate) for categorical variables. Multivariable logistic regression models were constructed to identify the covariates associated with proliferative LN. Covariates with a *p* value of < 0.05 in the univariable logistic regression analysis were incorporated to the multivariable models. In the multivariable analysis, the variable inflation factor was tested to exclude multicollinearity among covariates. The Hosmer-Lemeshow test was used to assess the goodness of fit for the logistic regression models. Antibodies and complements (C3 and C4) were analyzed as binary variables (positive/negative for antibodies, and low/not low for complements) in univariable analysis and multivariable analysis (model 1) and were analyzed as continuous variables in multivariable analysis (model 2). We used a receiver-operating characteristic (ROC) analysis to assess the ability of the covariates identified in the multivariable models in discriminating proliferative LN from membranous LN. ROC curves were generated, and the associated area under the curve (AUC) for each covariate was determined. The statistical significance level was set at a *p* value of < 0.05. All analyses were conducted using the SPSS software (version 25.0; IBM Corporation, Armonk, NY, USA).

### Sensitivity analysis

To test the robustness of our results, we performed several sensitivity analyses. First, we used a more restrictive definition of proliferative LN. Instead of including both pure proliferative LN (class III and class IV) and mixed proliferative LN (class III + V and class IV + V) in the proliferative LN group, we included only the pure proliferative LN (class III and class IV) in the proliferative LN group and performed multivariable logistic regression analysis and ROC analysis. Second, we compared mixed proliferative LN (class III + V and class IV + V) with membranous LN (class V). Third, as patients with classes I, II, and VI would also have to undergo a renal biopsy for diagnostic purposes, we included the patients with classes I, II, and VI and compared pure proliferative LN (class III and class IV) with non-proliferative LN (class I, class II, class V, and class VI).

## Results

### Patient characteristics

A total of 176 patients with biopsy-proven LN were included. The patients were predominantly women (90.9%), with a mean age of 36.7 ± 15.0 years. Among the patients, 150 patients (85.2%) had proliferative LN, and 18 patients (10.2%) had membranous LN. Of the 150 patients with proliferative LN, 122 (81.3%) patients had pure proliferative LN (class III, 38 patients; class IV, 84 patients) and 28 (18.7%) patients had mixed proliferative LN (class III + V, 17 patients; class IV + V, 11 patients) (Table 1). Three (1.7%) patients with class I, four (2.3%) patients with class II, and one (0.6%) patient with class VI were excluded for primary analysis.

**Table 1:** Histologic characteristics of the 176 patients

	*N* = 176
ISN/RPS class	
I, *n* (%)	3 (1.7%)
II, *n* (%)	4 (2.3%)
III, *n* (%)	38 (21.6%)
IV, *n* (%)	84 (47.7%)
III + V, *n* (%)	17 (9.7%)
IV + V, *n* (%)	11 (6.3%)
V, *n* (%)	18 (10.2%)
VI, *n* (%)	1 (0.6%)
Activity index, median (IQR)	7.0 (3.0–11.0)
Chronicity index median (IQR)	1.0 (0.5–2.5)

*ISN/RPS* International Society of Nephrology/Renal Pathology Society, *IQR* interquartile range

The comparison of the characteristics between the two groups is shown in Table 2. Age (*p* = 0.269), sex distribution (*p* = 0.649), and the proportion of patients with hypertension (*p* > 0.999) and diabetes mellitus (*p* = 0.599) did not differ between the two groups. The proportion of patients with mucocutaneous manifestations (*p* > 0.999), musculoskeletal manifestations (*p* = 0.408), neuropsychiatric manifestations (*p* = 0.149), and serositis (*p* = 0.475) was similar in the two groups; conversely, hematologic manifestations were more common in the patients with proliferative LN (44.7% vs 16.7%, *p* = 0.023). In the comparison of serologic covariates, no significant differences were observed in the positivity for anti-Sm Ab (*p* = 0.384), anti-Ro Ab (*p* = 0.885), anti-La Ab (*p* = 0.258), lupus anticoagulant (*p* = 0.768), anti-β_2_ glycoprotein Ab (*p* = 0.077), and anti-cardiolipin Ab (*p* = 0.566) and albumin levels (*p* = 0.800). The patients with proliferative LN were less commonly positive for anti-U1RNP Ab (48.7% vs 77.8%, *p* = 0.020) and more commonly positive for anti-dsDNA Ab (88.0% vs 50.0%, *p* < 0.001), and more commonly had low C3 (94.0% vs 61.1%, *p* < 0.001) and low C4 (79.3% vs 50.0%, *p* = 0.015), higher creatinine level (1.13 ± 0.79 mg/dL vs 0.70 ± 0.26 mg/dL, p < 0.001), and lower GFR (83.2 ± 37.0 mL/min/1.73 m^2^ vs 105.1 ± 23.8 mL/min/1.73 m^2^, *p* = 0.002). Regarding the urine laboratory data, the urine PCR (*p* = 0.778) and proportion of patients with pyuria (*p* = 0.053) and urine casts (*p* = 0.202) did not differ between the two groups. The proportion of patients with urine RBC of ≥ 5/HPF was higher in the proliferative LN group (72.7% vs 38.9%, *p* = 0.003). The SLEDAI-2 K was also higher in the proliferative LN group (17.1 ± 5.8 vs 12.2 ± 5.8, *p* = 0.001).

**Table 2:** Comparison of the clinical characteristics between the patients with proliferative LN and membranous LN

	Proliferative LN (*N* = 150)	Membranous LN (*N* = 18)	*p*
Age, mean (± SD), years	35.8 (± 14.3)	39.8 (± 15.4)	0.269
Female sex, *n* (%)	138 (92.0%)	16 (88.9%)	0.649
Hypertension, *n* (%)	34 (22.7%)	4 (22.2%)	> 0.999
Diabetes mellitus, *n* (%)	9 (6.0%)	0 (0.0%)	0.599
SLE manifestations			
Mucocutaneous, *n* (%)	41 (27.3%)	5 (27.8%)	> 0.999
Musculoskeletal, *n* (%)	41 (27.3%)	3 (16.7%)	0.408
Neuropsychiatric, *n* (%)	10 (6.7%)	3 (16.7%)	0.149
Serositis, *n* (%)	23 (15.3%)	1 (5.6%)	0.475
Hematologic, *n* (%)	67 (44.7%)	3 (16.7%)	0.023
Serology			
Positive anti-Sm Ab, *n* (%)	59 (39.3%)	9 (50.0%)	0.384
Positive anti-Ro Ab, *n* (%)	89 (59.3%)	11 (61.1%)	0.885
Positive anti-La Ab, *n* (%)	44 (29.3%)	3 (16.7%)	0.258
Positive anti-U1RNP Ab, *n* (%)	73 (48.7%)	14 (77.8%)	0.020
Positive anti-dsDNA Ab, *n* (%)	132 (88.0%)	9 (50.0%)	< 0.001
Anti-dsDNA Ab level, median (IQR), IU/mL	230.3 (79.0–380.0)	9.0 (2.5–123.5)	< 0.001
Positive anti-dsDNA Ab and negative anti-U1RNP Ab, *n* (%)	66 (44.0%)	1 (5.6%)	0.002
Positive anti-U1RNP Ab and negative anti-dsDNA Ab, *n* (%)	7 (4.7%)	6 (33.3%)	0.001
Positive anti-dsDNA Ab and anti-U1RNP Ab, *n* (%)	66 (44.0%)	8 (44.4%)	0.971
Positive lupus anticoagulant, *n* (%)	32 (21.3%)	3 (16.7%)	0.768
Positive anti-β_2_ glycoprotein I Ab, *n* (%)	18 (12.0%)	5 (27.8%)	0.077
Positive anti-cardiolipin Ab, *n* (%)	39 (26.0%)	3 (16.7%)	0.566
Low C3, *n* (%)	141 (94.0%)	11 (61.1%)	< 0.001
C3 level, median (IQR), mg/dL	41.0 (27.9–60.1)	64.7 (44.3–92.3)	0.001
Low C4, *n* (%)	119 (79.3%)	9 (50.0%)	0.015
C4 level, median (IQR), mg/dL	5.5 (2.5–11.9)	12.8 (7.3–23.5)	< 0.001
Albumin level, mean (± SD), g/dL	2.8 (± 0.7)	2.9 (± 0.9)	0.800
Creatinine level, mean (± SD), mg/dL	1.13 (± 0.79)	0.70 (± 0.26)	< 0.001
GFR, mean (± SD), mL/min/1.73 m^2^	83.2 (± 37.0)	105.1 (± 23.8)	0.002
Urine			
Urine PCR, median (IQR), mg/g	3935.0 (1903.4–6439.7)	3464.5 (2061.8–5775.0)	0.778
Urine RBC of ≥ 5/HPF, *n* (%)	109 (72.7%)	7 (38.9%)	0.003
Urine WBC of ≥ 5/HPF, *n* (%)	86 (57.3%)	6 (33.3%)	0.053
Urine cast, *n* (%)	31 (20.7%)	1 (5.6%)	0.202
SLEDAI-2K, mean (± SD)	17.1 (± 5.8)	12.2 (± 5.8)	0.001

*LN* lupus nephritis, *SLE* systemic lupus erythematosus, *Ab* antibody, *anti-dsDNA* anti-double-stranded DNA, *GFR* glomerular filtration rate, *PCR* protein/creatinine ratio, *RBC* red blood cell, *HPF* high power field, *WBC* white blood cell, *SLEDAI-2K* Systemic Lupus Erythematosus Disease Activity Index 2000, *SD* standard deviation, *IQR* interquartile range

### Covariates associated with proliferative LN

In the univariable logistic regression analysis, the presence of hematologic manifestations (unadjusted odds ratio [OR] = 4.036, 95% confidence interval [CI] = 1.121–14.527, *p* = 0.033), positive anti-dsDNA Ab (unadjusted OR = 7.333, 95% CI = 2.574–20.893, *p* < 0.001), low C3 (unadjusted OR = 9.970, 95% CI 3.117–31.891, *p* < 0.001), low C4 (unadjusted OR 3.839, 95% CI 1.405–10.486, *p* = 0.009), creatinine levels (unadjusted OR = 10.645, 95% CI = 1.350–83.953, *p* = 0.025), presence of urine RBC of ≥ 5/HPF (unadjusted OR = 4.718, 95% CI = 1.516–11.509, *p* = 0.006), and higher SLEDAI-2 K (unadjusted OR = 1.173, 95% CI = 1.063–1.294, *p* = 0.001) were associated with proliferative LN. The positivity of anti-U1RNP Ab (unadjusted OR = 0.271, 95% CI = 0.085–0.861, *p* = 0.027) and GFR (unadjusted OR = 0.980, 95% CI = 0.964–0.997, *p* = 0.021) were inversely associated with proliferative LN. These covariates were included in the multivariable models, except for the creatinine level, because of the multicollinearity with the GFR.

In multivariable analysis model 1, anti-U1RNP (positive/negative), anti-dsDNA Ab (positive/negative), C3 (low/not low), and C4 (low/not low) were analyzed as the categorical variables. An alternative multivariable analysis model (model 2) was also performed, in which anti-U1RNP, anti-dsDNA Ab, C3 level, and C4 level were analyzed as the continuous variables. Both the positivity (model 1) for and level (model 2) of anti-dsDNA Ab (model 1: adjusted OR = 11.200, 95% CI = 2.202–56.957, *p* = 0.004; model 2: adjusted OR = 1.008, 95% CI = 1.002–1.014, *p* = 0.014) were associated with proliferative LN; conversely, both the positivity (model 1) for and level (model 2) of anti-U1RNP Ab (model 1: adjusted OR = 0.176, 95% CI = 0.040–0.769, *p* = 0.021; model 2: adjusted OR = 0.985, 95% CI = 0.0976–0.994, *p* = 0.002) were inversely associated with proliferative LN. Higher GFR (model 1: adjusted OR = 0.973, 95% CI = 0.951–0.994, *p* = 0.013; model 2: adjusted OR = 0.969, 95% CI = 0.946–0.994, *p* = 0.014) was also inversely associated with proliferative LN (Table 3).

**Table 3:** Factors associated with proliferative LN

	Univariable analysis		Multivariable analysis (model 1)		Multivariable analysis (model 2)	
	OR (95% CI)	*p*	OR (95% CI)	*p*	OR (95% CI)	*p*
Age	0.982 (0.951–1.014)	0.269				
Female sex	1.437 (0.295–7.006)	0.653				
Hypertension	1.026 (0.317–3.322)	0.966				
Diabetes mellitus	N/A	0.999				
Mucocutaneous manifestations	0.978 (0.328–2.915)	0.968				
Musculoskeletal manifestations	1.881 (0.517–6.836)	0.337				
Neuropsychiatric manifestations	0.357 (0.088–1.442)	0.148				
Serositis	3.079 (0.390–24.280)	0.286				
Hematologic manifestations	4.036 (1.121–14.527)	0.033	3.277 (0.665–16.148)	0.145	1.963 (0.393–9.810)	0.411
Positive anti-Sm Ab	0.648 (0.243–1.728)	0.386				
Positive anti-Ro Ab	0.928 (0.341–2.529)	0.885				
Positive anti-La Ab	2.075 (0.572–7.528)	0.267				
Positive anti-U1RNP Ab^a^	0.271 (0.085–0.861)	0.027	0.176 (0.040–0.769)	0.021	0.985 (0.976–0.994)	0.002
Positive anti-dsDNA Ab^a^	7.333 (2.574–20.893)	< 0.001	11.200 (2.202–56.957)	0.004	1.008 (1.002–1.014)	0.014
Positive lupus anticoagulant	1.356 (0.370–4.974)	0.646				
Positive anti-β_2_ glycoprotein I Ab	0.355 (0.113–1.112)	0.075				
Positive anti-cardiolipin Ab	1.757 (0.483–6.396)	0.393				
Low C3^a^	9.970 (3.117–31.891)	< 0.001	1.886 (0.255–13.932)	0.534	1.012 (0.968–1.058)	0.608
Low C4^a^	3.839 (1.405–10.486)	0.009	1.224 (0.229–6.552)	0.813	0.929 (0.834–1.033)	0.175
Albumin level	0.886 (0.434–1.805)	0.738				
Creatinine level	10.645 (1.350–83.953)	0.025				
GFR	0.980 (0.964–0.997)	0.021	0.973 (0.951–0.994)	0.013	0.969 (0.946–0.994)	0.014
Urine PCR	1.000 (0.987–1.013)	0.986				
Urine RBC of ≥ 5/HPF	4.178 (1.516–11.509)	0.006	2.053 (0.426–9.893)	0.370	1.721 (0.307–9.652)	0.537
Urine WBC of ≥ 5/HPF	2.687 (0.958–7.543)	0.060				
Urine cast	4.429 (0.567–34.578)	0.156				
SLEDAI-2K	1.173 (1.063–1.294)	0.001	1.046 (0.900–1.216)	0.557	1.112 (0.923–1.338)	0.263

*Ab* antibody, *anti-dsDNA* anti-double-stranded DNA, *GFR* glomerular filtration rate, *PCR* protein/creatinine ratio, *RBC* red blood cell, *HPF* high power field, *WBC* white blood cell, *SLEDAI-2K* Systemic Lupus Erythematosus Disease Activity Index 2000, *OR* odds ratio, *CI* confidence interval, *N/A* not applicable

^a^Analyzed as binary variables (anti-U1RNP Ab, positive/negative; anti-dsDNA Ab, positive/negative; C3, low/not low; C4, low/not low) in univariable analysis and multivariable analysis (model 1) and analyzed as continuous variables in multivariable analysis (model 2)

### Ability of the covariates in discriminating proliferative LN

**Fig. 1:**
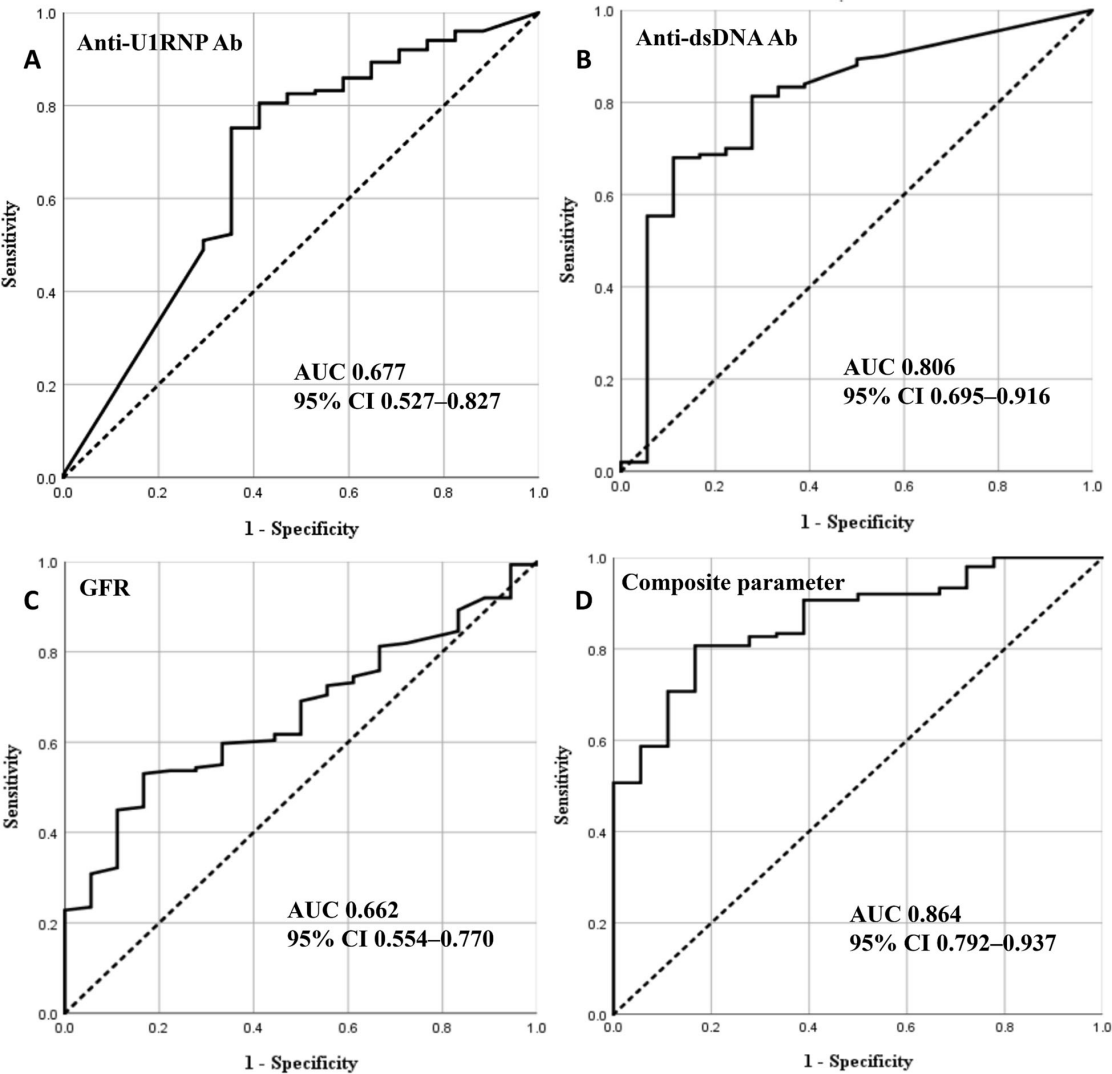
Receiver-operating characteristic curves for the predictive value of **a** anti-U1RNP Ab, **b** anti-dsDNA Ab, **c** GFR, and **d** combination of anti-U1RNP Ab, anti-dsDNA Ab, and GFR as a composite parameter, for predicting proliferative LN (class III, class IV, class III + V, and class IV + V). Ab, antibody; anti-dsDNA, anti-double-stranded DNA; GFR, glomerular filtration rate; LN, lupus nephritis; AUC, area under the curve; CI, confidence interval

The ROC curves for anti-U1RNP Ab, anti-dsDNA Ab, and the GFR are shown in Fig. 1. Anti-dsDNA Ab had the highest discrimination ability (AUC = 0.806, 95% CI = 0.695–0.916), followed by anti-U1RNP Ab (AUC = 0.677, 95% CI = 0.527–0.827) and GFR (AUC = 0.662, 95% CI = 0.554–0.770). When a combination of anti-dsDNA Ab, anti-U1RNP Ab, and GFR was used as a composite parameter, the discrimination ability (AUC = 0.864, 95% CI = 0.792–0.937) was higher than when each parameter was used as a single parameter (Fig. 1d).

### Sensitivity analysis

**Fig. 2:**
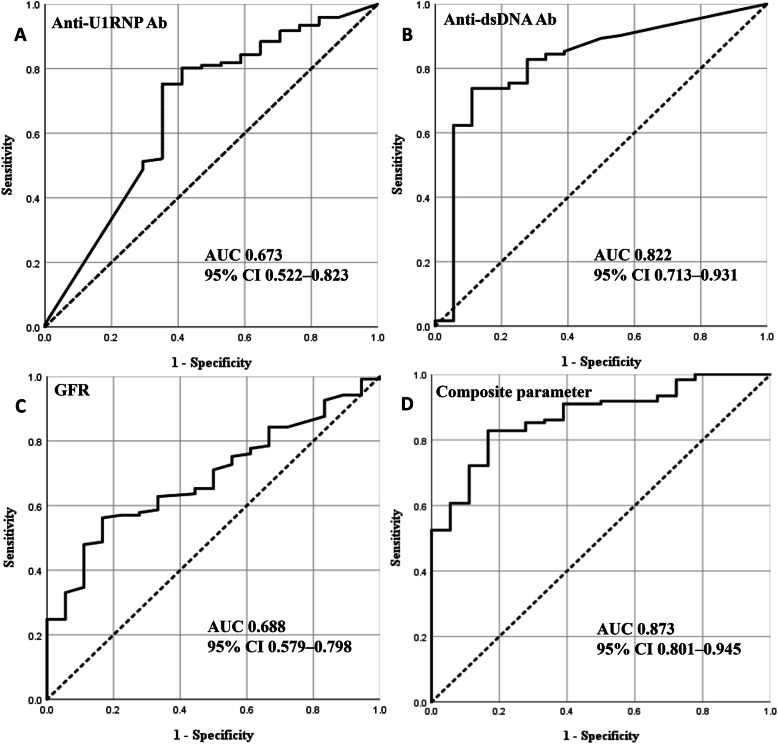
Receiver-operating characteristic curves for the predictive value of **a** anti-U1RNP Ab, **b** anti-dsDNA Ab, **c** GFR, and **d** combination of anti-U1RNP Ab, anti-dsDNA Ab, and GFR as a composite parameter, for predicting pure proliferative LN (class III and class IV). Ab, antibody; anti-dsDNA, anti-double-stranded DNA; GFR, glomerular filtration rate; LN, lupus nephritis; AUC, area under the curve; CI, confidence interval

When using the stricter proliferative LN definition (i.e., pure proliferative LN: class III and class IV), the ORs of anti-dsDNA Ab (model 1: adjusted OR = 19.591, 95% CI = 2.518–152.431, *p* = 0.004; model 2: adjusted OR = 1.008, 95% CI = 1.002–1.015, *p* = 0.012), anti-U1RNP Ab (model 1: adjusted OR = 0.178, 95% CI = 0.034–0.922, *p* = 0.040; model 2: adjusted OR = 0.987, 95% CI = 0.978–0.996, *p* = 0.007), and GFR (model 1: adjusted OR = 0.966, 95% CI = 0.941–0.992, *p* = 0.010; model 2: adjusted OR = 0.967, 95% CI = 0.942–0.992, *p* = 0.011) remained significant (Table 4). Further, the discrimination ability of anti-U1RNP Ab (AUC = 0.673, 95% CI = 0.522–0.823), anti-dsDNA Ab (AUC = 0.822, 95% CI = 0.713–0.931), GFR (AUC = 0.688, 95% CI = 0.579–0.798), and combination of anti-dsDNA Ab, anti-U1RNP Ab, and GFR as a composite parameter (AUC = 0.873, 95% CI = 0.801–0.945) was similar to the primary analysis (Fig. 2).

**Table 4:** Sensitivity analysis: factors associated with pure proliferative LN

	Univariable analysis		Multivariable analysis (model 1)		Multivariable analysis (model 2)	
	OR (95% CI)	*p*	OR (95% CI)	*p*	OR (95% CI)	*p*
Age	0.985 (0.954–1.017)	0.351				
Female sex	1.400 (0.281–6.976)	0.681				
Hypertension	1.043 (0.318–3.422)	0.945				
Diabetes mellitus	N/A	0.999				
Mucocutaneous manifestations	1.046 (0.347–3.153)	0.936				
Musculoskeletal manifestations	1.932 (0.526–7.097)	0.321				
Neuropsychiatric manifestations	0.304 (0.071–1.305)	0.109				
Serositis	3.535 (0.446–28.035)	0.232				
Hematologic manifestations	4.531 (1.248–16.453)	0.022	4.997 (0.819–30.479)	0.081	2.150 (0.408–11.333)	0.367
Positive anti-Sm Ab	0.718 (0.267–1.936)	0.513				
Positive anti-Ro Ab	1.051(0.381–2.904)	0.923				
Positive anti-La Ab	2.262 (0.618–8.279)	0.218				
Positive anti-U1RNP Ab^a^	0.268 (0.083–0.859)	0.027	0.178 (0.034–0.922)	0.040	0.987 (0.978–0.996)	0.007
Positive anti-dsDNA Ab^a^	8.385 (2.824–24.896)	< 0.001	19.591 (2.518–152.431)	0.004	1.008 (1.002–1.015)	0.012
Positive lupus anticoagulant	1.224 (0.328–4.572)	0.763				
Positive anti-β_2_ glycoprotein I Ab	0.337 (0.104–1.088)	0.069				
Positive anti-cardiolipin Ab	1.854 (0.504–6.819)	0.353				
Low C3^a^	14.891 (4.044–54.831)	< 0.001	2.216 (0.216–22.738)	0.503	1.004 (0.959–1.052)	0.866
Low C4^a^	5.421 (1.906–15.422)	0.002	1.763 (0.313–9.916)	0.520	0.947 (0.839–1.069)	0.376
Albumin level	0.898 (0.420–1.918)	0.781				
Creatinine level	11.857 (1.524–92.273)	0.018				
GFR	0.978 (0.961–0.995)	0.012	0.966 (0.941–0.992)	0.010	0.967 (0.942–0.992)	0.011
Urine PCR	0.999 (0.986–1.013)	0.934				
Urine RBC of ≥ 5/HPF	4.238 (1.515–11.852)	0.006	1.803 (0.358–9.087)	0.475	1.724 (0.299–9.952)	0.542
Urine WBC of ≥ 5/HPF	2.604 (0.917–7.391)	0.072				
Urine cast	4.381 (0.556–34.519)	0.161				
SLEDAI-2K	1.181 (1.067–1.308)	0.001	1.026 (0.884–1.190)	0.739	1.094 (0.899–1.330)	0.370

*Ab* antibody, *anti-dsDNA* anti-double-stranded DNA, *GFR* glomerular filtration rate, *PCR* protein/creatinine ratio, *RBC* red blood cell, *HPF* high power field, *WBC* white blood cell, *SLEDAI-2K* Systemic Lupus Erythematosus Disease Activity Index 2000, *OR* odds ratio, *CI* confidence interval, *N/A*, not applicable

^a^Analyzed as binary variables (anti-U1RNP Ab, positive/negative; anti-dsDNA Ab, positive/negative; C3, low/not low; C4, low/not low) in univariable analysis and multivariable analysis (model 1) and analyzed as continuous variables in multivariable analysis (model 2)

In another sensitivity analysis where mixed proliferative LN (class III + V and IV + V) was compared with membranous LN (class V), positivity of anti-dsDNA Ab was still significantly associated with mixed proliferative LN (model 1: adjusted OR = 4.545, 95% CI 1.107–18.661, *p* = 0.036) (Table 5), although the effect size was attenuated compared with the primary analysis (anti-dsDNA Ab in model 1: adjusted OR = 11.200) (Table 3) and in the sensitivity analysis where pure proliferative LN was compared with membranous LN (anti-dsDNA Ab in model 1: adjusted OR = 19.591) (Table 4). The effect size of anti-U1RNP Ab (unadjusted OR = 0.286, 95% CI 0.075–1.086, *p* = 0.066) and GFR (unadjusted OR = 0.988, 95% CI 0.968–1.009, *p* = 0.273) was also attenuated and failed to reach statistical significance (Table 5).

**Table 5:** Sensitivity analysis: factors associated with mixed proliferative LN (classes III + V and IV + V, *n* = 28) vs membranous LN (class V, *n* = 18)

	Univariable analysis		Multivariable analysis (model 1)		Multivariable analysis (model 2)	
	OR (95% CI)	*p*	OR (95% CI)	*p*	OR (95% CI)	*p*
Age	0.965 (0.920–1.012)	0.142				
Female sex	1.625 (0.208–12.705)	0.644				
Hypertension	0.955 (0.228–3.995)	0.949				
Diabetes mellitus	N/A	> 0.999				
Mucocutaneous manifestations	0.709 (0.180–2.792)	0.623				
Musculoskeletal manifestations	1.667 (0.370–7.515)	0.506				
Neuropsychiatric manifestations	0.600 (0.107–3.363)	0.561				
Serositis	1.308 (0.110–15.570)	0.832				
Hematologic manifestations	2.368 (0.544–10.317)	0.251				
Positive anti-Sm Ab	0.400 (0.116–1.376)	0.146				
Positive anti-Ro Ab	0.552 (0.165–1.838)	0.333				
Positive anti-La Ab	1.364 (0.294–6.319)	0.692				
Positive anti-U1RNP Ab^a^	0.286 (0.075–1.086)	0.066				
Positive anti-dsDNA Ab^b^	4.600 (1.207–17.524)	0.025	4.545 (1.107–18.661)	0.036	1.003 (0.999–1.007)	0.159
Positive lupus anticoagulant	2.000 (0.452–8.841)	0.361				
Positive anti-β_2_ glycoprotein I Ab	0.433 (0.099–1.900)	0.267				
Positive anti-cardiolipin Ab	1.364 (0.294–6.319)	0.692				
Low C3^a^	3.818 (0.922–15.808)	0.065				
Low C4^a^	1.333 (0.406–4.379)	0.635				
Albumin level	0.866 (0.435–1.721)	0.680				
Creatinine level	5.076 (0.446–57.756)	0.190				
GFR	0.988 (0.968–1.009)	0.273				
Urine PCR	1.002 (0.985–1.020)	0.808				
Urine RBC of ≥ 5/HPF	3.929 (1.122–13.755)	0.032	3.326 (0.559–19.795)	0.187	2.402 (0.464–12.428)	0.296
Urine WBC of ≥ 5/HPF	3.091 (0.895–10.672)	0.074				
Urine cast	4.636 (0.509–42.246)	0.174				
SLEDAI-2K	1.134 (1.015–1.266)	0.026	1.020 (0.872–1.195)	0.801	1.074 (0.935–1.233)	0.315

*Ab* antibody, *anti-dsDNA* anti-double-stranded DNA, *GFR* glomerular filtration rate, *PCR* protein/creatinine ratio, *RBC* red blood cell, *HPF* high power field, *WBC* white blood cell, *SLEDAI-2K* Systemic Lupus Erythematosus Disease Activity Index 2000, *OR* odds ratio, *CI* confidence interval, *N/A* not applicable

^a^Analyzed as binary variables (anti-U1RNP Ab, positive/negative; C3, low/not low; C4, low/not low) in univariable analysis

^b^Analyzed as binary variables (Anti-dsDNA Ab, positive/negative) in univariable analysis and multivariable analysis (model 1) and analyzed as continuous variables in multivariable analysis (model 2)

In comparison between pure proliferative LN (class III and class IV) and non-proliferative LN (class I, class II, class V, and class VI), anti-dsDNA Ab (model 1: adjusted OR = 13.741, 95% CI = 3.058–61.753, *p* = 0.001; model 2: adjusted OR = 1.008, 95% CI = 1.003–1.012, *p* = 0.002) was associated with pure proliferative LN, and anti-U1RNP Ab (model 1: adjusted OR = 0.273, 95% CI = 0.085–0.873, *p* = 0.029; model 2: adjusted OR = 0.991, 95% CI = 0.984–0.998, *p* = 0.012) and GFR (model 1: adjusted OR = 0.970, 95% CI = 0.952–0.989, *p* = 0.003; model 2: adjusted OR = 0.972, 95% CI = 0.954–0.990, p = 0.002) were inversely associated with pure proliferative LN, supporting their value in predicting proliferative LN (Table 6).

**Table 6:** Sensitivity analysis: factors associated with pure proliferative LN (classes III and IV, *n* = 122) vs non-proliferative LN (classes I, II, V and VI, *n* = 26)

	Univariable analysis		Multivariable analysis (model 1)		Multivariable analysis (model 2)	
	OR (95% CI)	*p*	OR (95% CI)	*p*	OR (95% CI)	*p*
Age	0.977 (0.951–1.002)	0.074				
Female sex	2.036 (0.586–7.082)	0.263				
Hypertension	0.809 (0.308–2.120)	0.666				
Diabetes mellitus	N/A	0.999				
Mucocutaneous manifestations	1.690 (0.591–4.834)	0.328				
Musculoskeletal manifestations	1.623 (0.566–4.649)	0.367				
Neuropsychiatric manifestations	0.335 (0.090–1.241)	0.102				
Serositis	2.495 (0.547–11.375)	0.238				
Hematologic manifestations	2.460 (0.964–6.276)	0.060				
Positive anti-Sm Ab	0.838 (0.358–1.962)	0.684				
Positive anti-Ro Ab	1.212 (0.513–2.863)	0.662				
Positive anti-La Ab	2.488 (0.802–7.719)	0.115				
Positive anti-U1RNP Ab^a^	0.345 (0.135–0.880)	0.026	0.273 (0.085–0.873)	0.029	0.991 (0.984–0.998)	0.012
Positive anti-dsDNA Ab^a^	8.385 (3.210–21.900)	< 0.001	13.741 (3.058–61.753)	0.001	1.008 (1.003–1.012)	0.002
Positive lupus anticoagulant	1.347 (0.424–4.276)	0.613				
Positive anti-β_2_ glycoprotein I Ab	0.544 (0.177–1.674)	0.289				
Positive anti-cardiolipin Ab	2.039 (0.654–6.362)	0.220				
Low C3^a^	10.400 (3.062–35.318)	< 0.001	2.072 (0.281–15.292)	0.475	0.994 (0.958–1.031)	0.747
Low C4^a^	3.975 (1.586–9.967)	0.003	1.811 (0.430–7.635)	0.418	0.958 (0.868–1.058)	0.395
Albumin level	0.665 (0.347–1.274)	0.218				
Creatinine level	2.722 (0.970–7.634)	0.057				
GFR	0.983 (0.969–0.996)	0.013	0.970 (0.952–0.989)	0.003	0.972 (0.954–0.990)	0.002
Urine PCR	1.003 (0.990–1.015)	0.669				
Urine RBC of ≥ 5/HPF	3.678 (1.534–8.819)	0.004	2.038 (0.538–7.720)	0.295	2.400 (0.588–9.796)	0.222
Urine WBC of ≥ 5/HPF	2.083 (0.875–4.959)	0.097				
Urine cast	1.976 (0.549–7.113)	0.297				
SLEDAI-2K	1.149 (1.056–1.251)	0.001	1.012 (0.896–1.144)	0.845	1.017 (0.883–1.171)	0.818

*Ab* antibody, *anti-dsDNA* anti-double-stranded DNA, *GFR* glomerular filtration rate, *PCR* protein/creatinine ratio, *RBC* red blood cell, *HPF* high power field, *WBC* white blood cell, *SLEDAI-2K* Systemic Lupus Erythematosus Disease Activity Index 2000, *OR* odds ratio, *CI* confidence interval, *N/A* not applicable

^a^Analyzed as binary variables (anti-U1RNP Ab, positive/negative; anti-dsDNA Ab, positive/negative; C3, low/not low; C4, low/not low) in univariable analysis and multivariable analysis (model 1) and analyzed as continuous variables in multivariable analysis (model 2)

## Discussion

In this retrospective cohort study, we showed that the positivity for and level of anti-dsDNA Ab were significantly associated with proliferative LN and that the positivity for and level of anti-U1RNP Ab and the GFR were inversely associated with proliferative LN. Among these covariates, anti-dsDNA Ab had the highest ability to discriminate proliferative LN from membranous LN. These findings are meaningful in that they may aid in therapeutic decision-making for clinicians when renal biopsies are difficult to perform.

Several previous studies have reported non-histologic factors associated with LN in patients with SLE [11–14]. Although there are some inconsistencies among these reports, anti-dsDNA Ab is consistently reported as an autoantibody that is associated with the occurrence of renal disease in SLE [11–14]. Mechanistically, anti-dsDNA Ab is involved in the development of LN by binding to glomerular and tubulointerstitial cells, inducing cell proliferation, inflammation, apoptosis, and fibrosis [15]. Similar to our present finding, a previous study also reported anti-dsDNA Ab as an important factor associated with proliferative LN compared with non-proliferative LN [16]. We further advanced the previous report by performing ROC analysis and providing the predictive value of anti-dsDNA Ab. Moreover, we also included anti-ENA Abs such as anti-Ro Ab, anti-La Ab, and anti-U1RNP Ab as variables in our analysis and found that anti-U1RNP Ab was inversely associated with proliferative LN, which was not reported in the previous study.

Anti-U1RNP Ab, which is by definition found in 100% of patients with mixed connective tissue disease, is found in 20–40% of patients with SLE [17]. Previous studies have reported an association between anti-U1RNP Ab and occurrence of pulmonary hypertension in patients with SLE [18–20]. There are conflicting data regarding the association between anti-U1RNP Ab and renal disease in SLE [11–13, 21]. One study reported a 66% reduced risk of LN development in patients with SLE with positive anti-U1RNP Ab findings [21], whereas other studies reported no association between the presence of anti-U1RNP Ab and LN development [11, 12] or even a higher risk of LN development [13]. Although the association between anti-U1RNP Ab and the presence of renal disease in SLE is controversial, the presence of anti-U1RNP Ab may have clinical significance when confined to patients with LN in that it is inversely associated with proliferative LN. In other words, the presence of anti-U1RNP Ab in patients with LN may suggest that the glomerular pathology might be membranous LN. This finding is meaningful because it is the first to indicate an association between anti-U1RNP Ab and the renal pathologic class.

We also found that the GFR was inversely associated with proliferative LN; a lower GFR was suggestive of proliferative LN. The other covariates associated with renal manifestations, such as amount of proteinuria and presence of hematuria, pyuria, and urine casts, were not associated with proliferative LN. Although these covariates are the components used in measuring disease activity (SLEDAI-2 K), they are not necessarily associated with a particular renal histology. Rather, the GFR was closely associated with the histologic classes of LN, although it is not a component of the SLEDAI-2 K.

Sensitivity analyses showed that effect sizes (i.e., ORs) of anti-dsDNA Ab, anti-U1RNP Ab, and GFR were greatest when pure proliferative LN was compared with membranous LN, followed by when proliferative LN (both pure and mixed proliferative LN) was compared with membranous LN, and when mixed proliferative LN was compared with membranous LN. This suggests that anti-dsDNA Ab, anti-U1RNP Ab, and GFR are particularly useful in detecting pure proliferative LN. Further, in the analysis where class I, class II, and class VI were included, anti-dsDNA Ab, anti-U1RNP Ab, and GFR were still significantly associated with pure proliferative LN, showing the robustness of the findings of the primary analysis.

We used the ROC analysis to assess the ability of anti-U1RNP Ab, anti-dsDNA Ab, and the GFR to predict proliferative LN. Given that the AUCs can be interpreted as follows: 1.00–0.90 = excellent, 0.80–0.90 = good, 0.70–0.80 = fair, 0.60–0.70 = poor, and 0.50–0.60 = fail [22], anti-dsDNA Ab (AUC = 0.806) had good accuracy, while anti-U1RNP Ab (AUC = 0.677) and the GFR (AUC = 0.662) had poor accuracy in discriminating between proliferative LN and membranous LN in the present study. When anti-dsDNA Ab was combined with anti-U1RNP Ab and GFR, the accuracy was numerically higher (AUC = 0.864) than when used as a single parameter. Similar results were also found in our sensitivity analysis. None of the covariates had an AUC of 1.00; therefore, these covariates cannot completely replace renal biopsy findings in discriminating between proliferative LN and membranous LN. However, in circumstances where renal biopsy is difficult to perform, anti-dsDNA Ab or its combination with anti-U1RNP Ab and GFR may be useful in discriminating between them, considering its good discriminating accuracy.

The present study has some limitations. First, as renal biopsies were performed only in the patients with overt clinical renal manifestations, patients with “silent” LN, which is defined as histologic LN in the absence of clinical renal manifestations [23, 24], were not included in our study. Therefore, our findings may not be generalized to patients with silent LN. However, considering that the value of renal biopsy and need for treatment is uncertain in silent LN [23], the covariates associated with proliferative LN in the patients with overt renal manifestations that we identified in this study still have clinical significance. Second, although we identified the covariates associated with proliferative LN, explanation for their associations cannot be drawn from the present study data. Further studies elucidating the mechanisms underlying these associations would be helpful. Third, the course of the study was long, and due to the retrospective nature of our study, we were unable to clarify whether the assay for measuring anti-dsDNA Ab has changed over time. Although the unit of anti-dsDNA Ab was the same (IU/ml) in all patients included, the potential of confounding remains by the possibility that the assay may have changed during the study period.

## Conclusion

In conclusion, we found that anti-U1RNP Ab, anti-dsDNA Ab, and the GFR are associated with glomerular pathology in patients with LN. Among these covariates, the anti-dsDNA Ab had a good accuracy in discriminating proliferative LN from membranous LN. Although anti-dsDNA Ab cannot replace the performance of renal biopsy findings, it can be helpful in patients with SLE with clinically overt renal manifestations who cannot undergo renal biopsies.

## Data Availability

All data generated or analyzed during this study are included in this article.

## Abbreviations

LN: Lupus nephritis
SLE: Systemic lupus erythematosus
ESRD: End-stage renal disease
ACR: American College of Rheumatology
PCR: Protein/creatinine ratio
RBC: Red blood cell
HPF: High power field
Ab: Antibody
anti-dsDNA: Anti-double-stranded DNA
GFR: Glomerular filtration rate
SLEDAI-2 K: Systemic lupus erythematosus disease activity index 2000
ROC: Receiver-operating characteristic
AUC: Area under the curve
OR: Odds ratio
CI: Confidence interval
